# Uric acid and arterial stiffness in children and adolescents: Role of insulin resistance and blood pressure

**DOI:** 10.3389/fcvm.2022.978366

**Published:** 2022-08-11

**Authors:** Simonetta Genovesi, Laura Montelisciani, Francesca Viazzi, Marco Giussani, Giulia Lieti, Ilenia Patti, Antonina Orlando, Laura Antolini, Paolo Salvi, Gianfranco Parati

**Affiliations:** ^1^School of Medicine and Surgery, University of Milano-Bicocca, Milan, Italy; ^2^Cardiology Unit, Istituto Auxologico Italiano, IRCCS, Milan, Italy; ^3^Department of Internal Medicine, University of Study and IRCCS Ospedale Policlinico San Martino, Genova, Italy

**Keywords:** pulse wave velocity, children, uric acid, insulin resistance, blood pressure, mediation analysis

## Abstract

Several studies describe the association between serum uric acid (SUA) and arterial stiffness in adults. Uric acid contributes through several mechanisms to the increase in blood pressure (BP) and adversely affects the insulin signaling pathway. Moreover, SUA predict the development of hypertension and insulin resistance up to type 2 diabetes. Early arterial stiffening, estimated by carotid-femoral pulse wave velocity (PWV), may already be present in pediatric age. Aim of our study was to investigate the relationship between SUA and PWV in a pediatric population and its interaction with insulin resistance and BP. In 322 children and adolescents (56.2% male, mean age 11.3 [SD 2.8] years), we measured weight, height, waist circumference, BP and PWV. We also assayed SUA and estimated glomerular filtration rate (eGFR) and calculated HOMA-index as a marker of insulin resistance. Simple and multiple regression analyses were performed to assess variables associated with PWV. Mediation models were applied to identify the direct and indirect effects of individual variables on PWV. On univariate analysis, age (*p* < 0.001), waist circumference-to-height ratio (*p* = 0.036), systolic and diastolic BP (SBP and DBP) *z-*score (*p* < 0.001), heart rate (*p* = 0.028), SUA (*p* = 0.002), HOMA-index (*p* < 0.001), and eGFR (*p* = 0.014) were significantly associated with PWV. The multiple regression model showed that only age (*p* = 0.028), SBP *z-*score (*p* = 0.006), and heart rate (*p* = 0.001) were significantly associated with PWV. The results were superimposable when the DBP *z-*score replaced the SBP *z-*score in the model. Mediation models showed that the effect of eGFR on PWV was fully mediated by SUA (*p* = 0.015) and that the effect of SUA on PWV was totally mediated by HOMA-index (*p* < 0.001). Both SUA (*p* < 0.01) and HOMA-index (*p* < 0.01) had a significant association with higher SBP (DBP) *z-*scores. The double mediation model including both BP and HOMA-index showed that the SUA effect on PWV was totally mediated by both variables (*p* = 0.005, for HOMA-index, *p* = 0.004, for SBP *z-*score and *p* = 0.007, for combined effect). The results were superimposable when the DBP *z-*score replaced the SBP *z-*score in the model. In conclusion, insulin resistance and BP are both important mediators of the association between SUA and vascular stiffness in pediatric age.

## Introduction

Hyperuricemia and insulin resistance (IR) are well known risk factors for hypertension (HT) (1, 2), even in children (3). While many studies have found that the relationship between serum uric acid (SUA) and the development of HT is weakened after adjusting for IR (4–6), some data support the role of IR in mediating the effect of increased SUA on the risk of developing HT suggesting that SUA may be a causal factor of IR (7).

Longitudinal studies performed in adults show that SUA and IR influence blood pressure (BP) and the risk of cardiovascular events (8, 9). This process is probably driven by several mechanisms including (a) activation of the renin angiotensin aldosterone system, (b) increase in renal sodium reabsorption, and (c) reduction of nitric oxide generation leading to vascular dysfunction (10–14).

Serum uric acid and homeostatic model assessment (HOMA) index, a recognized indicator of IR, are also associated with vascular damage in adulthood (3). Due to the major impact of even subclinical organ damage as a risk factor for cardiovascular diseases, it is important to clarify the possible role of BP and IR in mediating the effect of SUA on cardiovascular damage. While, in general, such damage occurs in adulthood, its development can start as early as during the first decade of life (15).

Carotid–femoral pulse wave velocity (PWV) is the non-invasive gold standard measurement of arterial stiffness (16), and it is associated with the incidence and severity of HT in adults. In children however, a close relationship between high BP and increased PWV has been described as well (17).

While a joint effect of increased SUA and IR on the development of subclinical vascular damage has been described in adults (18), it has not yet been completely elucidated whether these conditions synergistically interact for promoting vascular dysfunction also in children.

The aim of the study was to investigate the complex relationship between SUA levels, IR, BP and subclinical organ damage in children and adolescents at increased cardiovascular risk. In the attempt to optimize our methodological approach, in addition to classical statistical regression models, we used a mediation analysis, an efficient method to clarify the causal paths underlying the association between the explanatory factors and the outcome.

## Methods

### Subjects

We studied a cohort of 322 children and adolescents (3.7–18.4 years), consecutively referred from 06/08/2012 to 10/06/2020 to our Unit for Cardiovascular Risk Assessment in Children (at the Istituto Auxologico Italiano, Milan, Italy) by their primary care pediatricians, because of evidence of elevated BP values and/or excess weight. Exclusion criteria were: diabetes mellitus, any form of secondary HT and treatment with antihypertensive drugs.

The study protocol was approved by the local institutional ethics committee and conformed to the ethical guidelines of the 1975 Declaration of Helsinki (RICARPE study, 2015102002). An informed consent was obtained from parents or legal representatives before the enrolment in the study.

### Anthropometric parameters

In all children height, weight and waist circumference (WC) were measured. Weight was approximated to the nearest 100 grams, and height to the nearest 1 mm. Waist circumference was measured to the nearest 0.5 cm by a flexible tape in standing position. Body mass index (BMI) was calculated as weight (kg)/height (m)^2^. Waist-to-height-ratio (WtHr) was calculated dividing WC by height and expressed as percentage. BMI *z-*scores were calculated using the Centre for Disease and Control prevention charts available at https://www.cdc.gov/growthcharts/clinical_charts.htm. Weight class was defined according to the International Obesity Task Force classification (19) distinguishing among normal weight (NW), overweight (OW) and obese (OB) classes. Pubertal stage was assessed by a medical examination and children were classified into two categories: pre-pubertal and pubertal according to Tanner (20), considering pre-pubertal boys with gonadal stage 1 and girls with breast stage 1.

### Blood pressure assessment

Blood pressure was measured using an oscillometric device validated in children (Omron 705IT; Omron Co, Kyoto, Japan) with the appropriate cuff for the children's upper-arm size.

Blood pressure measurements were performed after at least 5 min of rest, with the child in a sitting position. The BP measurement was performed three times (at intervals of a few minutes) and the average of the last two measurements was considered. Systolic BP (SBP) and diastolic BP (DBP) percentiles and *z-*scores were calculated according to the nomograms of the National High Blood Pressure Education Program (NHBPEP) Working Group on High Blood Pressure in Children and Adolescents (21). The children were classified as follows: normotensive if both SBP and DBP percentiles were <90th; high-normal if SBP and/or DBP percentiles were ≥90th, but both <95th; hypertensive if SBP and/or DBP percentiles were ≥95th.

### Biochemical parameters

Blood samples were taken from all subjects after a 12-h fasting period to measure serum concentrations of glucose, insulin, SUA and creatinine. Commercial kits, normally used for routine examinations of patients, were employed for all analyses. In detail: enzymatic method with hexokinase Glucose HK Gen.3 Cobas Roche, for glucose assay; immuno Assay in ElectroChemiLuminescence Elecsys Insulin Cobas Roche for insulin assay; colorimetric enzymatic test Uric Acid 2 Cobas Roche for uric acid assay; colorimetric kinetic test based on the Jaffé method Creatinine Jaffé Gen.2 Cobas Roche for creatinine assay. HOMA-index was calculated by dividing the product of serum insulin (μU/ml) and serum glucose (mmol/L) by 22.5 (22). Glomerular filtration rate was estimated (eGFR) by means of the Schwartz formula using serum creatinine and height measurements and a k constant of 0.55 (23).

### Measurement of arterial distensibility

Measurements were obtained in a stable room temperature after 10 min of rest. A validated, easy-to-use and high-fidelity PulsePen tonometer (DiaTecne srl, San Donato Milanese, Italy) was used in this study (24, 25). Reproducibility of the PulsePen device (26) and the procedure has been described in detail previously (24, 27). Briefly, the PulsePen consists of a pocket size, high-fidelity applanation tonometer, and an integrated ECG unit. Aortic PWV was measured by recording carotid and femoral waveforms in rapid succession. Carotid-femoral PWV was defined as 80% of the distance between the measuring sites (28) divided by the time delay between the distal (femoral) pulse wave from the proximal (carotid) pulse wave, using the R wave of the ECG trace as reference. The PulsePen device software did not validate measurements if the difference between BP or heart rate values taken at the time of carotid and femoral artery recordings was >10%. The use of the PulsePen device in children had been validated in a previous study, which provided reference values for cf-PWV in children and adolescents (29).

## Statistical methods

Continuous variables were expressed as mean and standard deviation, the categorical variables as percentages on relative frequencies. Comparisons across strata, defined by gender, were obtained by *T*-test and Chi-square test.

The relationship between the outcome (PWV) and explanatory variables was investigated through univariate and multivariable linear regression models.

A mediation analysis through Structural Equation Modeling (SEM) was then performed to more deeply explore the roles of explanatory variables as predictors and possible mediators of PWV. Structural Equation Modeling is a very powerful multivariate technique that allows to examine a set of relationships among a few variables at the same time. It is based on a system of linked regression-style equations. In a classic regression model framework, there is a clear distinction between dependent and independent variables. In a SEM framework, however, a dependent variable in one model equation can become an independent variable in other components of the SEM system (30). Relationships are represented in a path diagram with nodes representing the variables and arrows showing the relations among the variables.

The mediation model was applied according to the following strategy. We explored the possible presence of an impact of SUA, mediated by HOMA-index, on PWV. Similarly, we explored the possible presence of an impact of HOMA-index, mediated by SUA, on PWV. The same approach was used to explore a possible mediation between SUA and HOMA-index on SBP and DBP and of SUA and eGFR on PWV. Suggested mediation paths were then included in a comprehensive model of PWV.

## Results

Three hundred and twenty-two children and adolescents (mean age 11.3 years, standard deviation [SD] 2.8 years; 56.2% males) were enrolled. Table 1 shows the main anthropometric and clinical characteristics of the study sample. A total of 156 (48.6%) individuals had begun pubertal development. Percentages of NW, OW and OB were 19.9, 30.4, 49.7%, respectively. Waist-to-height-ratio was greater or equal to 50% in 71.0%. Regarding BP category, a percentage of 72.7% was normotensive, 9.6% had high-normal BP values, and 17.7% was hypertensive. The mean of PWV values was 4.8 m/s (SD 0.97 m/s).

**Table 1:** Anthropometric and clinical characteristics of the study population.

**Variable**	**Total**		**Male**		**Female**		***p-*value**
	**(*n =* 322; 100.00%)**		**(*n =* 181; 56.21%)**		**(*n =* 141; 43.79%)**		
	**mean**	**SD**	**mean**	**SD**	**mean**	**SD**	
Age (years)	11.30	2.81	11.62	2.68	10.89	2.93	0.022
	***n*** **(%)**	SD	***n*** **(%)**	SD	***n*** **(%)**	SD	
Puberty Yes	156 (48.60)	0.03	81 (45.00)	0.04	75 (51.19)	0.40	0.145
Weight (kg)	55.96	19.91	59.63	20.98	51.26	17.42	<0.001
Height (m)	1.48	0.16	1.52	0.17	1.44	0.15	<0.001
BMI (kg/m^2^)	24.66	5.04	25.11	5.24	24.08	4.74	0.069
BMI *z-*score	1.53	0.96	1.57	0.99	1.47	0.92	0.401
Weight class	***n*** **(%)**	SD	***n*** **(%)**	SD	***n*** **(%)**	SD	0.733
Normal Weight	64 (19.88)	0.02	34 (18.78)	0.03	30 (21.28)	0.03	
Over Weight	98 (30.43)	0.03	58 (32.04)	0.03	40 (28.37)	0.04	
Obese	160 (49.69)	0.03	89 (49.17)	0.04	71 (50.35)	0.04	
WC (cm)	78.82	13.48	81.45	14.14	75.42	11.77	<0.001
WtHr (%)	53.17	7.13	53.71	7.22	52.48	6.99	0.131
WtHr>50%	225 (70.98)	0.03	134 (74.86)	0.03	91 (65.94)	0.04	0.083
SBP office (mmHg)	112.17	14.89	113.52	14.60	110.43	15.13	0.064
DBP office (mmHg)	66.12	9.72	65.90	9.05	66.40	10.56	0.645
SBP (z-score)	0.61	1.05	0.59	1.03	0.63	1.09	0.742
DBP (z-score)	0.36	0.77	0.31	0.72	0.43	0.82	0.155
Blood Pressure Category	***n*** **(%)**	SD	***n*** **(%)**	SD	***n*** **(%)**	SD	0.668
Normotensive	236 (72.67)	0.02	134 (74.03)	0.03	100 (70.92)	0.04	
High Normal	31 (9.63)	0.02	18 (9.94)	0.02	13 (9.22)	0.02	
Hypertensive	57 (17.70)	0.02	29 (16.02)	0.03	28 (19.86)	0.03	
Creatinine (mg/dl)	0.56	0.14	0.58	0.14	0.54	0.13	0.002
eGFR (ml/min)	149.63	25.57	147.44	24.57	152.44	26.62	0.082
Uric acid (mg/dl)	4.52	1.19	4.66	1.32	4.35	0.97	0.021
Glucose (mg/dl)	85.18	7.55	85.91	6.68	84.25	8.46	0.050
Insuli*n* (mM/L)	15.35	11.33	15.12	10.24	15.65	12.62	0.680
HOMA-index	3.29	2.54	3.26	2.34	3.31	2.78	0.864
PulseWaveVelocity (m/sec)	4.80	0.97	4.76	0.93	4.84	1.02	0.490
Heart rate (beats/min)	76.95	12.17	74.27	11.72	80.38	11.90	<0.001

*BMI, body mass index; WC, waist circumference; WtHr, waist circumference height ratio; SBP, systolic blood pressure; DBP, diastolic blood pressure; eGFR, estimated glomerular filtration rate. HOMA, homeostatic model assessment index*.

Univariable and multivariable linear regression models on PWV are shown in Table 2. Variables associated with PWV values by the univariable model were: age (*p* < 0.001), onset of pubertal developmental (*p* = 0.002), WtHr % (*p* = 0.036), SBP and DBP *z-*scores (*p* < 0.001), and heart rate (*p* = 0.028). There was a significant and positive association between PWV values and SUA (*p* = 0.002) and HOMA-index (*p* < 0.001), while the relationship between PWV and eGFR was inverse (*p* = 0.014).

**Table 2:** Univariable and multivariable linear regression model on pulse wave velocity (m/s) – (Model A adjusted by SBP and Model B adjusted by DBP).

	**Univariable**							**Multivariable—Model A**							**Multivariable - Model B**						
**Variable**	**b**	**(95% CI)**					***p*-value**	**b**	**(95% CI)**					***p*-value**	**b**	**(95% CI)**					***p*-value**
Intercept	–		–		–		–	2.836	(	1.309	;	4.362	)	<0.001	2.764	(	1.251	;	4.277	)	<0.001
Age (years)	0.085	(	0.049	;	0.122	)	**<0.001**	0.067	(	0.007	;	0.128	)	**0.028**	0.078	(	0.019	;	0.136	)	**0.010**
Gender (male)	−0.075	(	−0.289	;	0.139	)	0.490	–		–		–		–	–		–		–		–
Puberty (Yes)	0.333	(	0.123	;	0.543	)	**0.002**	−0.033	(	−0.328	;	0.262	)	0.826	−0.019	(	−0.314	;	0.276	)	0.898
Weight (kg)	0.012	(	0.007	;	0.018	)	**<0.001**	–		–		–		–	–		–		–		–
Height (m)	1.480	(	0.845	;	2.116	)	**<0.001**	–		–		–		–	–		–		–		–
BMI (kg/m^2^)	0.042	(	0.021	;	0.062	)	**<0.001**	–		–		–		–	–		–		–		–
BMI *z-*score	0.093	(	−0.018	;	0.203	)	0.099	–		–		–		–	–		–		–		–
Weight class							0.071	–		–		–		–	–		–		–		–
Normal Weight	ref.		–		–		–	–		–		–		–	–		–		–		–
Overweight	0.356	(	0.051	;	0.660	)		–		–		–		–	–		–		–		–
Obese	0.236	(	−0.044	;	0.516	)		–		–		–		–	–		–		–		–
WC (cm)	0.018	(	0.010	;	0.026	)	**<0.001**	–		–		–		–	–		–		–		–
WtHr (%)	0.016	(	0.001	;	0.031	)	**0.036**	0.010	(	−0.006	;	0.027	)	0.227	0.009	(	−0.008	;	0.026	)	0.307
WtHr > 50%	0.166	(	−0.071	;	0.403	)	0.168	–		–		–		–	–		–		–		–
SBP office (mmHg)	0.022	(	0.015	;	0.028	)	**<0.001**	–		–		–		–	–		–		–		–
DBP office (mmHg)	0.032	(	0.022	;	0.042	)	**<0.001**	–		–		–		–	–		–		–		–
SBP (*z*-score)	0.272	(	0.175	;	0.368	)	**<0.001**	0.156	(	0.046	;	0.267	)	**0.006**	–		–		–		–
DBP (*z*-score)	0.324	(	0.190	;	0.458	)	**<0.001**	–		–		–		–	0.209	(	0.063	;	0.355	)	**0.005**
Blood Pressure Category							**<0.001**	–		–		–		–	–		–		–		–
Normotensive	ref.		–		–			–		–		–		–	–		–		–		–
High Normal	0.377	(	0.023	;	0.073	)		–		–		–		–	–		–		–		–
Hypertensive	0.579	(	0.306	;	0.853	)		–		–		–		–	–		–		–		–
Creatinine (mg/dl)	1.414	(	0.643	;	2.184	)	**<0.001**	–		–		–		–	–		–		–		–
eGFR (ml/min)	−0.005	(	0.009	;	−0.001	)	**0.014**	−0.004	(	−0.009	;	0.001	)	0.086	−0.004	(	−0.008	;	0.001	)	0.116
Uric acid (mg/dl)	0.138	(	0.050	;	0.226	)	**0.002**	−0.020	(	−0.126	;	0.086	)	0.716	−0.018	(	−0.124	;	0.088	)	0.742
Glucose (mg/dl)	0.014	(	0.001	;	0.028	)	**0.045**	–		–		–		–	–		–		–		–
Insuli*n* (mM/L)	0.019	(	0.010	;	0.028	)	**<0.001**	–		–		–		–	–		–		–		–
HOMA–index	0.085	(	0.044	;	0.126	)	**<0.001**	0.041	(	−0.007	;	0.088	)	0.095	0.043	(	−0.005	;	0.091	)	0.076
Heart rate	0.010	(	0.001	;	0.018	)	**0.028**	0.015	(	0.006	;	0.024	)	**0.001**	0.015	(	0.006	;	0.024	)	**0.002**

*b, coefficient; CI, confidence interval; ref., reference; BMI, body mass index; WC, waist circumference; WtHr, waist circumference height ratio; SBP, systolic blood pressure; DBP, diastolic blood pressure: eGFR, estimated glomerular filtration rate. HOMA, homeostatic model assessment index. Statistically significant p-values are in bold*.

In the multivariable regression analysis including SBP *z-*scores, the variables that were shown to be significantly associated to PWV values were: age (*p* = 0.028), SBP *z-*scores (*p* = 0.006), and heart rate (*p* = 0.001) (Table 2, Model A). When DBP *z-*scores were included in the model instead of SBP *z-*scores, variables associated with PWV values were: age (*p* = 0.010), DBP *z-*scores (*p* = 0.005), and heart rate (*p* = 0.002) (Table 2, Model B).

**Figure 1:**
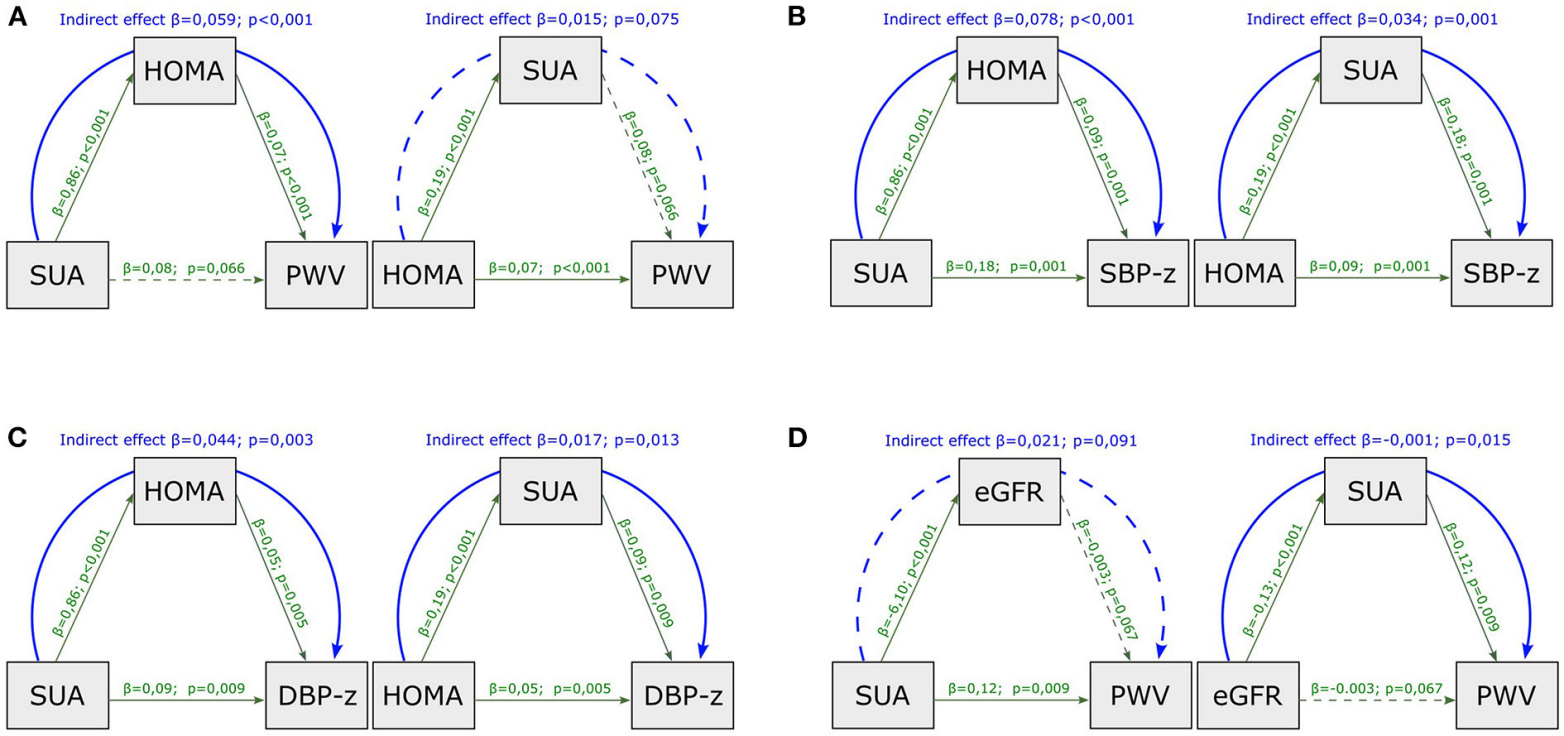
Mediation analysis model with PWV as outcome, including HOMA-index and SUA as mediators **(A)**, with SBP **(B)** and DBP **(C)** *z-*scores as outcome, including HOMA-index and SUA as mediators and with PWV as outcome including SUA and eGFR as mediators **(D)**. SUA, serum uric acid; HOMA-index, homeostatic model assessment index; PWV, pulse wave velocity; eGFR, estimated glomerular filtration rate; SBP-z, systolic blood pressure *z-*score; DBP-z, diastolic blood pressure *z-*score. Continuous line, statistically significant; dotted line, not statistically significant; green line, direct effect; blue line, indirect effect.

In the mediation analysis with PWV as outcome (Figure 1A), the effect of SUA was entirely mediated by HOMA-index (indirect effect, *p* < 0.001, Figure 1A); by contrast, the effect of HOMA-index on PWV was not mediated by SUA (indirect effect, *p* = 0.075).

When considering SBP *z-*scores and DBP *z-*scores as outcomes instead of PWV, HOMA-index and SUA showed both significant direct and indirect effects in the causal paths (Figures 1B,C).

In the mediation analysis with PWV as outcome (Figure 1D), the impact of eGFR was entirely mediated by SUA (indirect effect, *p* = 0.015), whereas the effect of SUA on PWV was not mediated by eGFR (indirect effect, *p* = 0.091).

**Figure 2:**
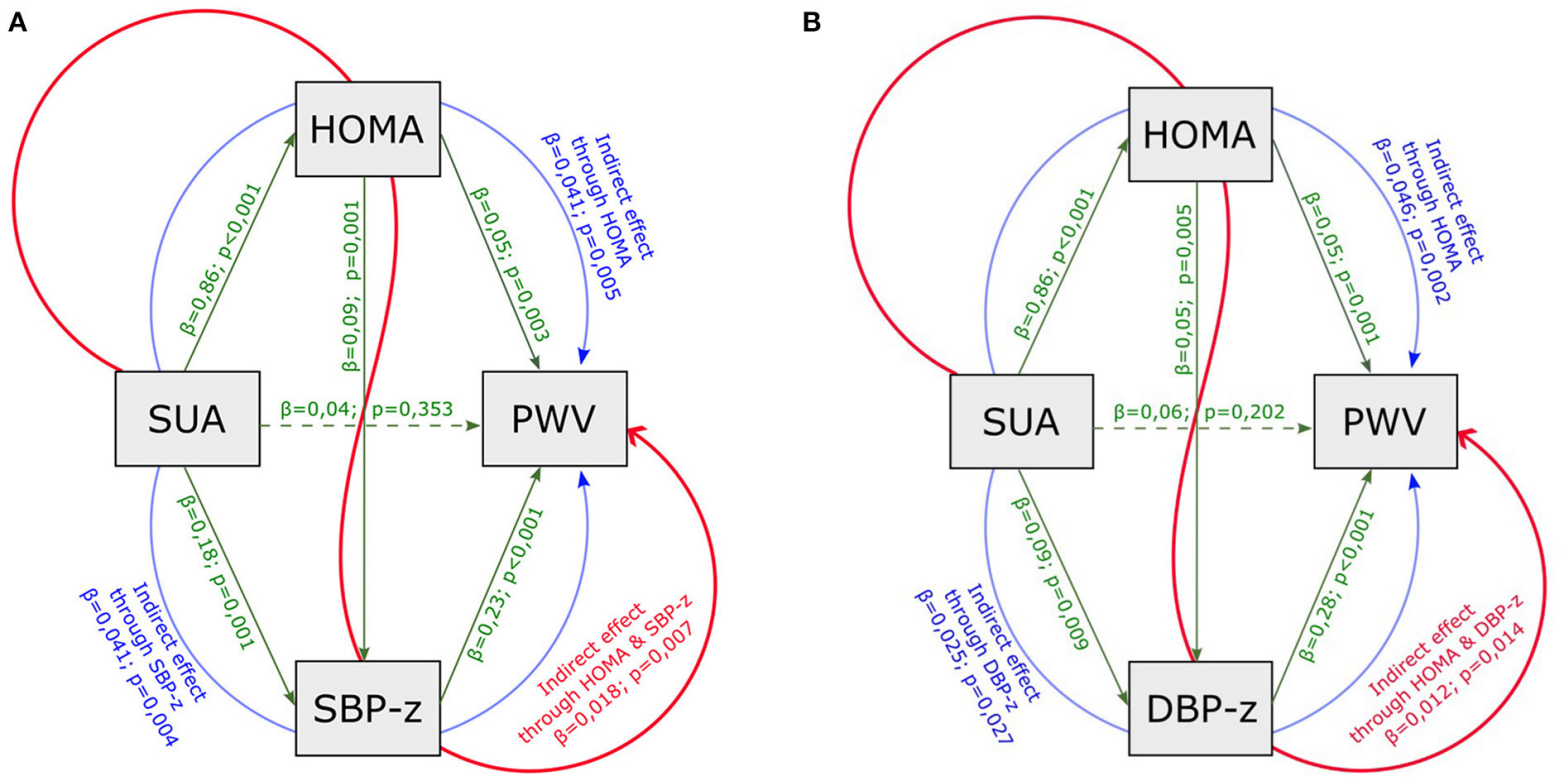
Mediation analysis model with PWV as outcome, including HOMA-index, SUA, SBP **(A)** and DBP **(B)** *z-*scores as mediators. SUA, serum uric acid; HOMA-index, homeostatic model assessment index; PWV, pulse wave velocity; eGFR, estimated glomerular filtration rate; SBP-z, systolic blood pressure *z-*score; DBP-z, diastolic blood pressure *z-*score. Continuous line, statistically significant; dotted line, not statistically significant; green line, direct effect; blue line, partial indirect effect (one mediator); red line, partial indirect effect (two mediators). Overall indirect effect (with SBP z) = 0,100 *p* < 0,001. Overall indirect effect (with DBP z) = 0,084 *p* < 0,001.

Based on the results shown, a double mediation model on PWV was carried out considering SUA as initial explanatory variable and including HOMA-index and SBP (DBP) *z-*score as mediators (Figures 2A,B). In this model the indirect effect of SUA was partitioned in three paths: the first involved HOMA-index only, the second involved SBP (DBP) only, the third involved both HOMA-index and SBP (DBP) *z-*score. This enlarged model confirmed the indirect effect of SUA through HOMA-index and both SBP and DBP *z-*scores, and the absence of a significant direct effect of SUA.

Similar results were obtained including the direct impact of other confounders on PWV (e.g., eGFR, gender, heart rate and age) and on SUA (eGFR) (Supplementary Figures 1, 2A,B).

## Discussion

Our study shows that, in a population of children and adolescents with high BP and/or excess weight, IR (quantified by the HOMA-index) and SUA values are strongly associated with increased aortic stiffness, as assessed by carotid-femoral PWV. This relationship seems to be lost when the statistical model is corrected for possible confounding factors. In fact, at classical multiple regression analysis, only BP (SBP and DBP *z-*scores), age, and heart rate maintain a significant association with PWV values. However, when the data are analyzed using mediation analysis, IR maintains an effect on vascular stiffness that is independent of SBP and DBP, while the association between SUA and PWV values turn out to be totally mediated by HOMA-index and BP.

These findings could account for all intervention studies that fail to demonstrate a significant efficacy of urate lowering treatment in terms of cardiovascular and renal protection (31). In fact, if the effect exerted by SUA on organ damage and therefore on events is largely mediated by its effect on the increase in BP and on the development of IR, SUA lowering treatment is unlikely to improve cardiovascular prognosis once these clinical conditions and the organ damage have already developed. Both in adults and children/adolescents a close association between PWV and BP values has been shown (17, 32). Hypertension is characterized by functional and structural changes leading to increased peripheral vascular resistance. On the other hand, endothelial dysfunction contributes to vascular remodeling of resistance arteries implicated in the development and complications of hypertension itself (14). As it has been proposed that increased PWV might represent an early vascular alteration that is already present at the onset of HT development (33), assessment of PWV has been included as a method for the identification of HT-related subclinical organ damage in the guidelines for the diagnosis and management of HT in adults (34).

In addition to BP levels, several clinical variables and clinical conditions such as heart rate, age, gender, presence of diabetes, atherosclerosis and kidney disease have been associated to increased PWV, both in adult and in young people (35–37). Our study confirms and extends data from the literature (38) describing the independent association between BP levels and arterial stiffness in a relatively large cohort of children and adolescents at increased cardiovascular risk.

Our results regarding the relationship between vascular stiffness and IR agree with those of other authors. Carotid-femoral PWV, evaluated in 315 healthy prepubertal children, was significantly correlated with HOMA-IR independently from 24-h BP levels and adiposity indices (39) and similar results were observed in a sample of prepubescent children with metabolically healthy obesity (40).

Hyperuricemia is commonly associated with HT and clinical conditions characterized by IR such as obesity and type 2 diabetes. In adulthood, elevated SUA levels are associated with an increased risk of incident HT, and it has been suggested that IR may mediate the relationship between SUA and HT (2). Therefore, previous studies investigated the complex relationship between vascular stiffness and SUA taking into consideration the intermediate role of IR or HT. Cassano et al. suggested that SUA and HOMA-index have a synergistic action in increasing vascular stiffness in a population of untreated hypertensive adults, showing that SUA and IR exerted their effect independently of each other and of BP values (18).

Increased SUA is strongly associated with increased HOMA-index in children (41); nevertheless, while IR is potentially implicated in the development of HT, the association between SUA and BP levels has been described to be independent from HOMA-index in a population of 501 children at cardiovascular risk (42).

Both cross-sectional (43, 44) and longitudinal studies (45, 46) that evaluated whether there is an independent association between SUA and arterial stiffness, yielded inconclusive results in adults. A recent meta-analysis showed an association between SUA and carotid-femoral PWV in a general population, not confirmed by 4 studies performed in 1225 hypertensive subjects (47).

While arterial stiffness has been investigated as a function of IR and BP dysfunction in children and adolescents, data on the relationship between SUA and arterial stiffness in pediatric age are few. In a sample of 188 adolescents affected by type 1 diabetes, no association could be observed between arterial stiffness and SUA values, suggesting that SUA seems not to be associated with vascular abnormalities in this clinical setting (48). Accordingly, Mocnik et al. (49) did not find any associations between SUA and arterial stiffness in a cohort of 269 hypertensive adolescents and young adults at increased risk for subclinical atherosclerosis. In the Lytvyn et al. study (47), the lack of association between SUA and PWV might be due to the stimulatory effect of urinary glucose on the GLUT9 in the proximal tubule which increases uric acid excretion in diabetes (50). In addition, in the study by Mocnik and al. (48), a subgroup of patients were taking antihypertensive. While the negative results described in these studies might be due to a selection bias, it is also possible that the complex interrelation between the variables investigated has conditioned the result, masking its true meaning. In a population consisting of 961 healthy adults and 570 children (>6 years old), the relationship between SUA values at baseline and after a 20-year follow-up on the incidence of HT and carotid-femoral PWV values was investigated. A significant association was present between SUA at baseline and incidence of HT at follow-up in both the adult-only and total population, whereas the association between SUA at baseline and higher PWV values at follow-up was significant only when children were included in the analysis. However, the study lacks an analysis performed only in the subgroup of children (51).

To better understand our results, and to find out whether the effects of any of the variables considered were mediated by those attributable to another variable, in addition to performing a classic uni- and multivariate analyses, we made use of mediation analysis. The mediation analysis is a method that can be applied to explain the causal paths underlying the association between the exposure and the outcome. If the mediators are on the causal path, then the exposure affects the mediator, which in turn affects the outcome. The total effect of exposure can pass both through the mediators (indirect effect) and through a direct pathway (direct effect). The mediation analysis makes it possible to identify the proportion of the direct and indirect effects of the exposure on the outcome. If the mediators fully mediate the effect from the exposure to the outcome, it is a full mediation case. Alternatively, situations where the mediators do not fully mediate the effect of the exposure on the outcome, represent partial mediation cases. In the present study the mediation analysis showed that both SUA and HOMA-index had a direct effect on SBP and DBP *z-*scores, while SUA and HOMA-index themselves had a reciprocal effect on each other. However, the effect of SUA on arterial stiffness appeared to be completely mediated by the level of IR, whereas HOMA-index had a direct effect on PWV. These observations were confirmed even after correction for other confounding factors (gender, age, heart rate and eGFR). The distribution of risk factors, but especially the time these risk factors had to exert their negative effect, varies with increasing age of children. It is very likely that the metabolic risk factors considered in the study, even if present very early in life, exert their negative effect to an increasing extent as the time since their onset increases. In the present study, age is significantly associated with higher PWV values in the multivariable regression model. To try to mitigate this drawback, we performed an age adjustment of the final mediation model. Age maintains its impact on PWV values, however, the main results of the study remain unchanged. Importantly, eGFR, which is strongly inversely related to SUA values, does not appear to have a direct effect on PWV levels, but only an indirect effect mediated by SUA, which, in turn, exerts its indirect effect on PWV through the HOMA-index. This result is probably because none of the children of the study had chronic kidney disease. Therefore, while the inverse relationship between eGFR and SUA is present even with normal renal function, the impact that a reduction in eGFR might have on the development of organ damage is determined by factors (e.g., an initial expansion of extracellular volume) that begin to act when renal damage is clinically evident.

Our study has some limitations as well as several strengths. Among the first ones, other factors that we did not consider in this study may alter endothelial function (e.g., inflammatory pathways, endothelin and nitric oxide) and may play an important role in determining vascular stiffness in this age group (14, 52). On the other hand, although assessing cardiovascular risk factors in young people precludes the demonstration of their associations with cardiovascular events, it still enabled us to evaluate the interaction of increased SUA values, high BP and markers of IR in determining vascular phenotypes, avoiding indirect and confounding effects related to the presence of comorbidities comorbidities. Moreover the use of carotid-femoral PWV appeared to be more effective in exploring the impact of several risk factors on vascular damage compared to the brachial-ankle PWV (53), perhaps because in adolescents brachial-ankle PWV is more strongly associated with the increase in cardiac output rather than with the increase of collagen and decrease of elastin. Finally, at variance with previous studies performed in adolescents, data from our analyses are more reliable due to the indexed values of BP (z-score) we applied instead of the raw data, commonly used in adults.

In conclusion, the interactions between BP, IR and SUA on one side and vascular stiffness on the other side are extremely complex, and a statistical analysis performed with classical methods (multiple regression analysis) is likely to miss this complexity. Our data suggest that SUA through the effects of IR and BP may cause structural remodeling of the macrovascular wall, which may further trigger arteriolar narrowing and increased large artery stiffness. The subclinical alterations of the vascular phenotype may play a key role in mediating the incidence of deleterious cardiovascular outcomes later in life. For this reason, PWV may be useful as a non-invasive tool to identify the presence of arterial stiffness in young people at increased cardiovascular risk. The presence of insulin resistance and hyperuricemia are not always considered important cardiovascular risk factors in children. Our study shows that these two metabolic factors are not only associated with the presence of HT in children and adolescents, but are also mediators of the onset of early organ damage, such as increased vascular stiffness, both directly (HOMA index) and indirectly (SUA). These findings should suggest the need for interventions aimed at reducing HOMA index and SUA values as early as pediatric age. Nevertheless, the results of our study contribute to the comprehension of the relationship between SUA levels, BP, IR and the occurrence of subclinical organ damage, without the chance to give indications about the threshold levels at which starting treatment strategies might be useful for primary prevention. It is therefore important to study potential preclinical mechanisms that lead to early disease pathogenesis to facilitate the identification of high-risk patients and thereby implement early therapeutic prevention strategies. Being able to analyze these phenomena in a pediatric population prior to the onset of clinical complications represents a very important because it allows to avoid some confounding factors such as vascular aging, drug interference, or smoking. Indeed, investigating changes observed in children offers a fascinating model for understanding the early pathophysiological mechanisms underlying HT.

## Data availability statement

The raw data supporting the conclusions of this article will be made available by the authors, without undue reservation.

## Ethics statement

The studies involving human participants were reviewed and approved by Ethic Committee Name: Istituto Auxologico Italiano, Approval Code: 2015102002, Approval Date: 20/11/2015. Written informed consent to participate in this study was provided by the participants' legal guardian/next of kin.

## Author contributions

SG conceptualized and designed the study, drafted the initial manuscript, and reviewed and revised the manuscript. IP, GL, AO, and MG collected data. LM and LA performed data analysis. GP, PS, and FV reviewed and revised the manuscript. All authors approved the final manuscript as submitted and agree to be accountable for all aspects of the work.

## Funding

Research funded by the Italian Ministry of Health.

## Conflict of interest

The authors declare that the research was conducted in the absence of any commercial or financial relationships that could be construed as a potential conflict of interest.

## Publisher's note

All claims expressed in this article are solely those of the authors and do not necessarily represent those of their affiliated organizations, or those of the publisher, the editors and the reviewers. Any product that may be evaluated in this article, or claim that may be made by its manufacturer, is not guaranteed or endorsed by the publisher.
