# Supplementary material for: Uric acid and arterial stiffness in children and adolescents: Role of insulin resistance and blood pressure
Source: Front Cardiovasc Med. 2022 Aug 11;9:978366. doi: 10.3389/fcvm.2022.978366 (PMC9403185; doi:10.3389/fcvm.2022.978366)
Supplement: Supplementary file 1 [file Data_Sheet_1.pdf]

Supplementary Figure 1

Mediation analysis model with PWV as outcome, including HOMA-index, SUA, SBP (panel a) and DBP (panel b) z-scores as mediators adjusted by heart rate and age

Panel A

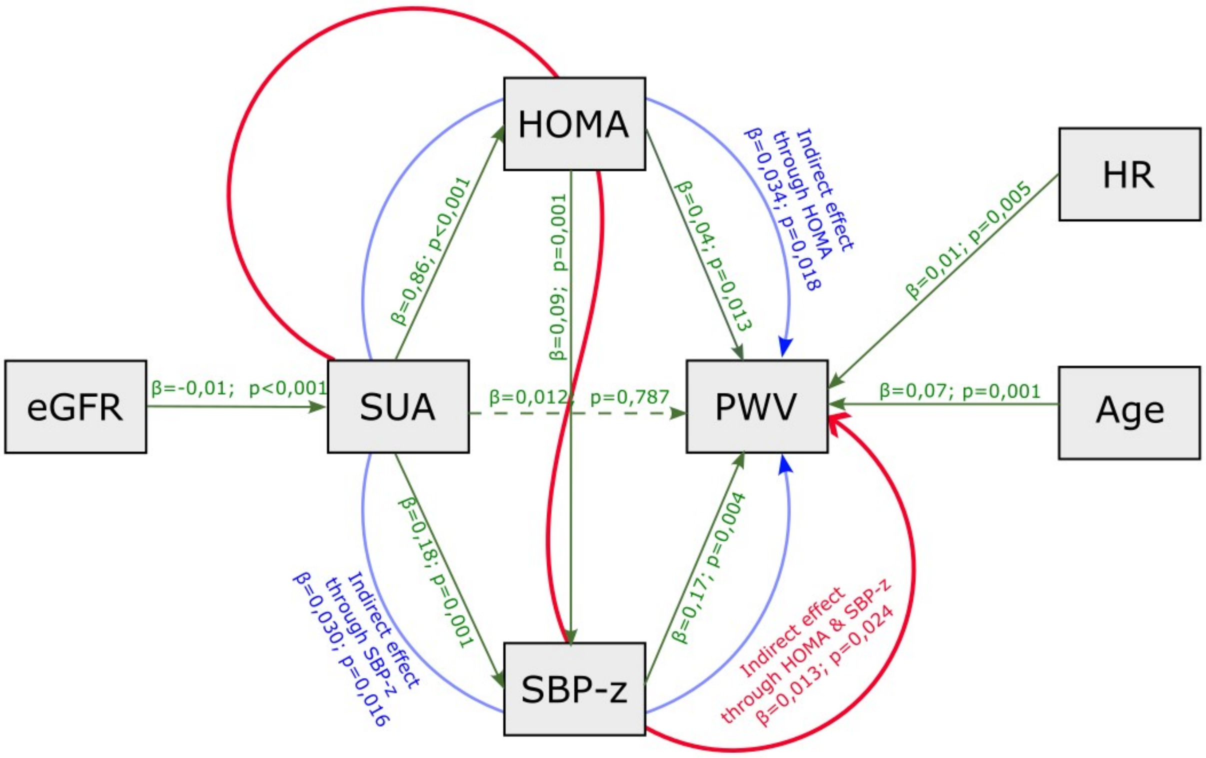

Panel B

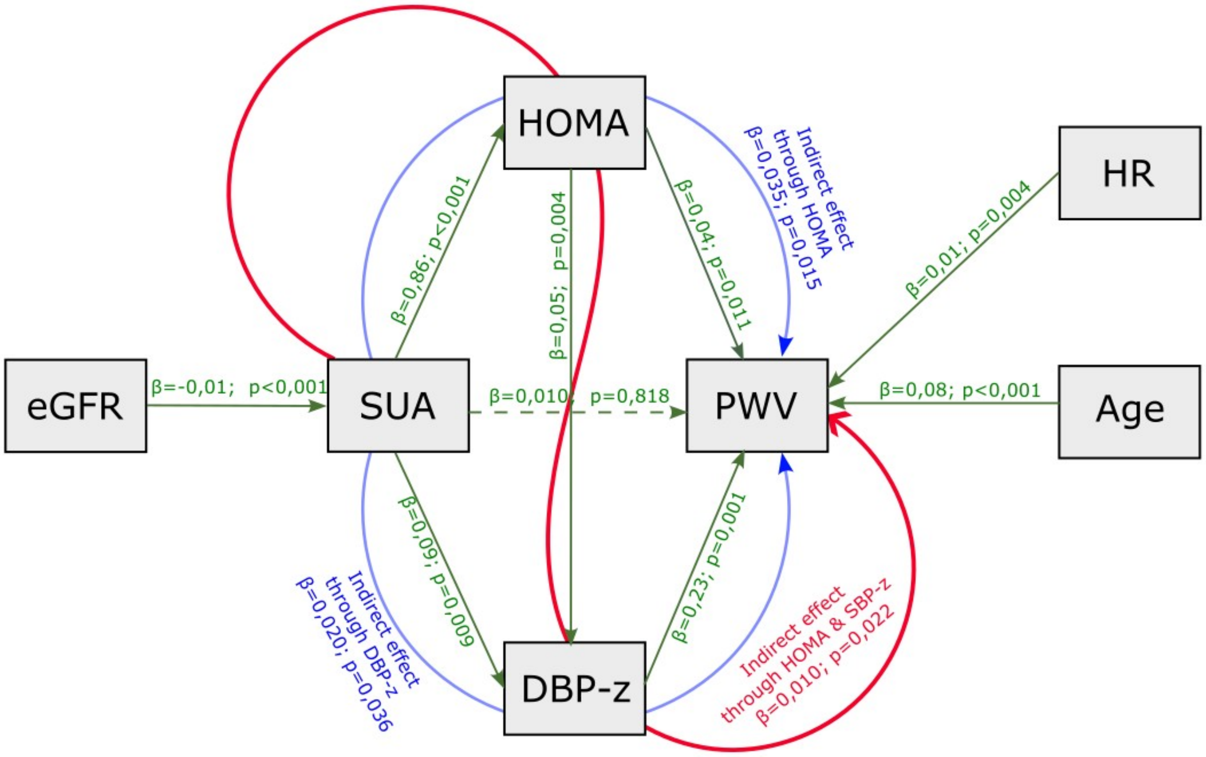

SUA = serum uric acid, HOMA = homeostatic model assessment index, PWV = pulse wave velocity, eGFR = estimated glomerular filtration rate, SBP z = systolic blood pressure z score, DBP z = diastolic blood pressure z score, HR = heart rate.

Full line = statistically significant; dotted line = not statistically significant.

Green line = direct effect, blue line = partial indirect effect (one mediator), red line = partial indirect effect (two mediators).

Overall indirect effect (with SBP z) = 0.077 p < 0,001.

Overall indirect effect (with DBP z) = 0.066 p < 0,001.

Supplementary Figure 2

Mediation analysis model with PWV as outcome, including HOMA-index, SUA, SBP (panel a) and DBP (panel b) z-scores as mediators adjusted by heart rate, age and gender

Panel A

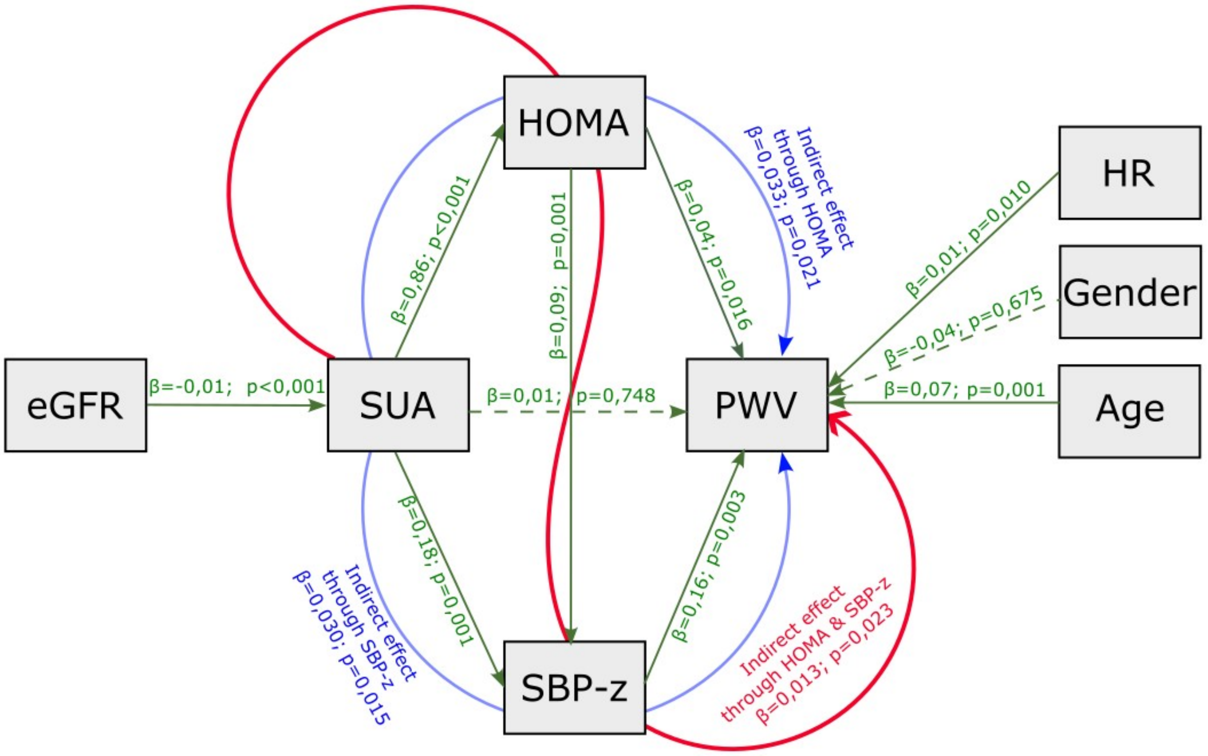

Panel B

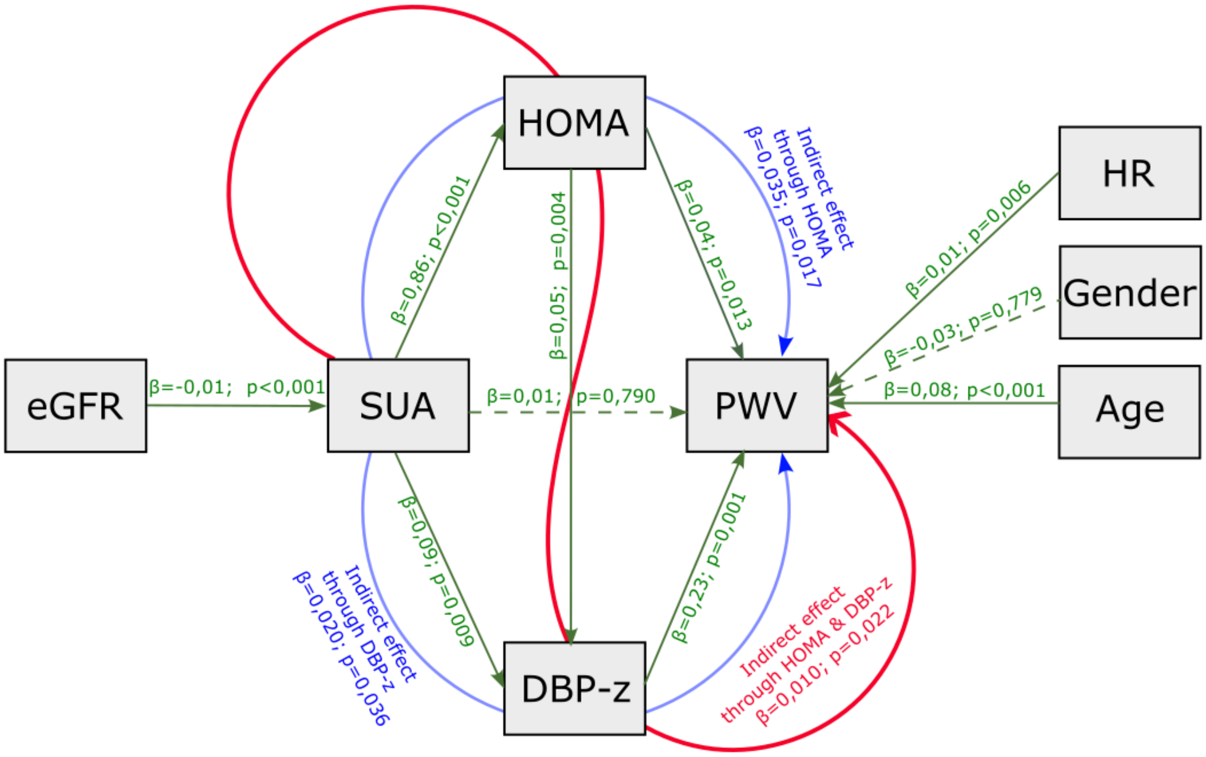

SUA = serum uric acid, HOMA = homeostatic model assessment index, PWV = pulse wave velocity, eGFR = estimated glomerular filtration rate, SBP z = systolic blood pressure z score, DBP z = diastolic blood pressure z score, HR = heart rate.

Full line = statistically significant; dotted line = not statistically significant.

Green line = direct effect, blue line = partial indirect effect (one mediator), red line = partial indirect effect (two mediators).

Overall indirect effect (with SBP z) = 0.076 p < 0,001.

Overall indirect effect (with DBP z) = 0.065 p < 0,001.
